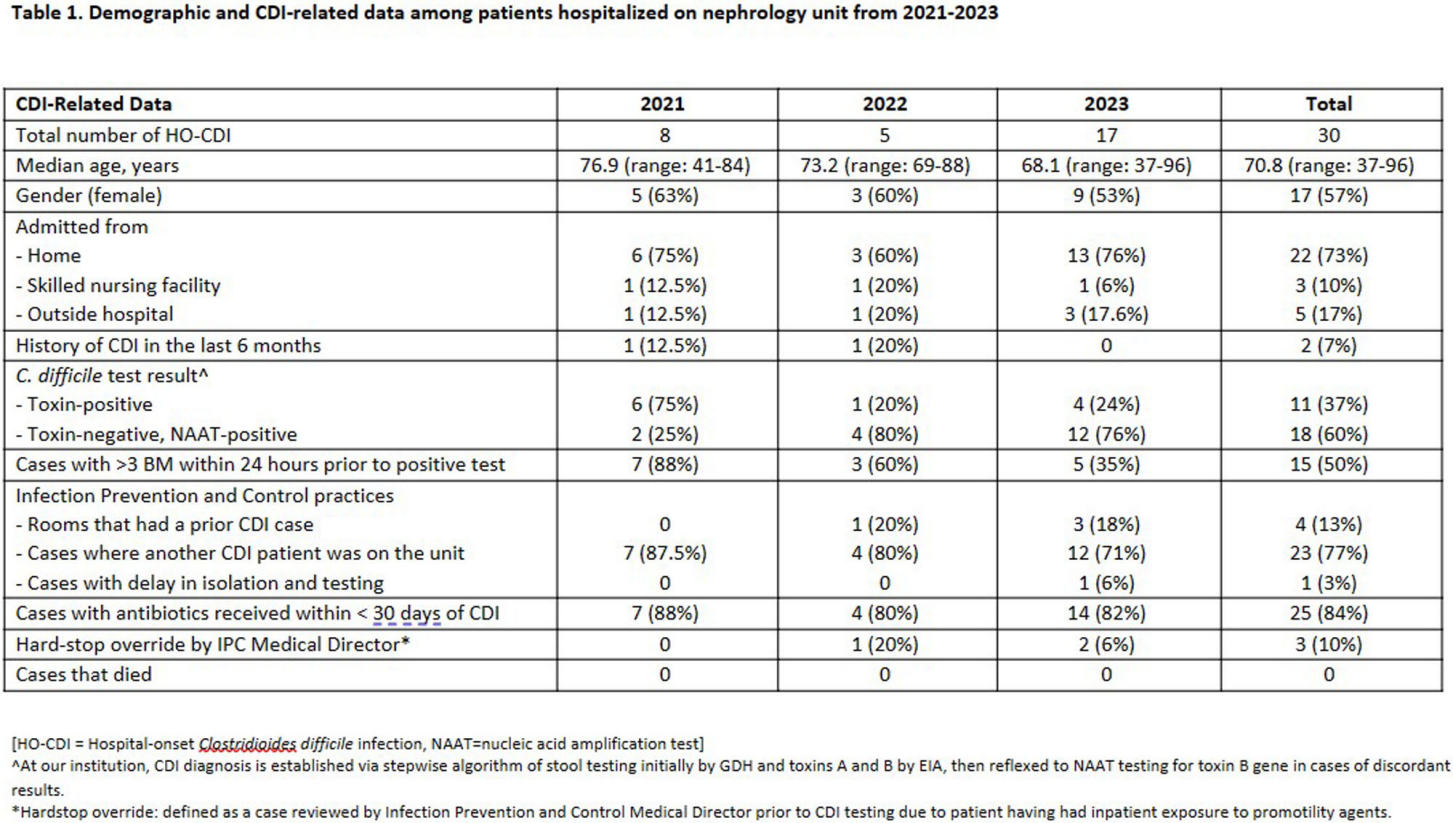# Hospital-Onset Clostridioides difficile infection in chronic kidney disease patients

**DOI:** 10.1017/ash.2024.193

**Published:** 2024-09-16

**Authors:** Anita Shallal, Clare Shanahan, Abigail Ruby, Eman Chami, Rachel Kenney, Geehan Suleyman

**Affiliations:** Henry Ford Hospital; Henry Ford Health System

## Abstract

**Introduction:** Hospital-onset Clostridioides difficile infection (HO-CDI), reported as laboratory-identified (LabID) event, is common in patients with chronic kidney disease (CKD), especially those with end-stage renal disease (ESRD), and is associated with prolonged length of hospitalization and more severe disease. CKD patients are at increased of developing CDI due to frequent antimicrobial and healthcare exposures. The objective of this study was to assess recent trends of HO-CDI in patients on a nephrology unit at our academic, tertiary care institution. **Methods:** Retrospective cross-sectional study of patients with HO-CDI who were hospitalized on a nephrology unit between January 2021 to December 2023. Collected variables included: demographic data, characterization of HO-CDI risk factors, infection and diagnosis (including prior history of CDI, toxin versus nucleic acid amplification test [NAAT] positivity, number of loose stools), CDI rate (defined as CDI count/patient days x1000), standardized antimicrobial administration ratio (SAAR) for high-risk for CDI antimicrobials (defined by the National Healthcare Safety Network), and infection prevention and control (IPC) practices, including hand hygiene audit rates. **Results:** A total of 30 HO-CDI infections were reported on the nephrology unit [Table], with 8 occurring in 2021, 5 in 2022, and 17 in 2023. The median age of patients was 70.8 (range: 37-96) years, and most patients (57%) were female. The majority of patients were admitted from home (73%), and two patients (7%) had a history of CDI in the last 6 months. Among the CDI cases, 60% were NAAT positive and toxin negative, and only 50% had >3 bowel movements (BM) within 24 hours prior to the positive test. Ten percent received promotility agents prior to testing. Most cases (77%) occurred when other CDI patients were on the unit. Hand hygiene compliance rates averaged 81% over the three-year period [Figure 1A]. Eight-four per cent of patients received antibiotics within 30 days of CDI diagnosis; SAAR was >1 for quarters 2 and 4 in 2022, and quarter 1 in 2023 [Figure 1B]. **Conclusion:** On our nephrology unit, patients often had < 3 BM within 24 hours of CDI diagnosis, and 60% of cases were toxin-negative, NAAT-positive, suggesting possible C. difficile colonization, rather than true infection. In addition, an elevated SAAR correlated with high CDI rates. Multicomponent interventions may be required to reduce the rates of HO-CDI in CKD patients. Opportunities include emphasis on diagnostic and antimicrobial stewardship, environmental cleaning and adherence to IPC practices, including hand hygiene.

**Figure:**
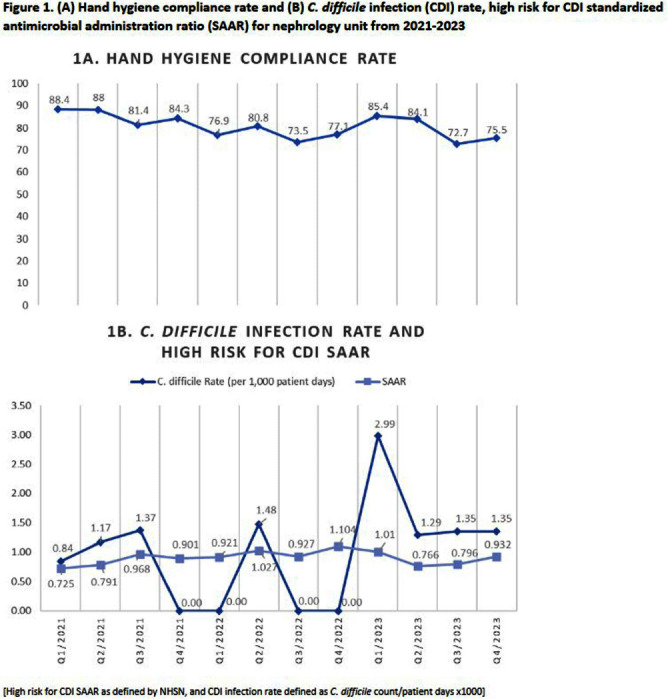

**Figure:**